# Sirtuin 3 is essential for host defense against *Mycobacterium abscessus* infection through regulation of mitochondrial homeostasis

**DOI:** 10.1080/21505594.2020.1809961

**Published:** 2020-09-09

**Authors:** Young Jae Kim, Sang-Hee Lee, Sang Min Jeon, Prashanta Silwal, Ju-Young Seo, Bui Thi Bich Hanh, June-Woo Park, Jake Whang, Min Joung Lee, Jun Young Heo, Soon Ha Kim, Jin-Man Kim, Gyu Yong Song, Jichan Jang, Eun-Kyeong Jo

**Affiliations:** a Department of Microbiology, Chungnam National University College of Medicine, Daejeon, Korea; b Infection Control Convergence Research Center, Chungnam National University College of Medicine, Daejeon, Korea; c Center for Research Equipment, Korea Basic Science Institute, Cheongju, Chungbuk, South Korea; d College of Pharmacy, Chungnam National University, Daejeon, Republic of Korea; e Molecular Mechanisms of Antibiotics, Division of Life Science, Research Institute of Life Science, Gyeongsang National University, Jinju, Korea; f Division of Applied Life Science (Bk21plus Program), Gyeongsang National University, Jinju, Korea; g Department of Environmental Toxicology and Chemistry, Korea Institute of Toxicology, Jinju, Korea; h Human and Environmental Toxicology Program, Korea University of Science and Technology (UST), Daejeon, Korea; i Korea Mycobacterium Resource Center (KMRC) & Basic Research Section, The Korean Institute of Tuberculosis (KIT) 168-5, Cheongju-si, Chungcheongbuk-do, Republic of Korea; j Department of Biochemistry, Chungnam National University College of Medicine, Korea; k Department of Medical Science, Chungnam National University College of Medicine, Daejeon, Korea; l MitoImmune Therapeutics, Inc., Ganhnam-gu, Seoul, Korea; m Department of Pathology; Chungnam National University College of Medicine, Korea

**Keywords:** *Mycobacterium abscessus*, sirtuin 3, mitochondrial reactive oxygen species, resveratrol, host-directed therapy

## Abstract

The global incidence of *Mycobacterium abscessus* (Mabc), a rapidly growing nontuberculous mycobacterial strain that causes treatment-refractory pulmonary diseases, is increasing. Despite this, the host factors that allow for protection against infection are largely unknown. In this study, we found that sirtuin 3 (SIRT3), a mitochondrial protein deacetylase, plays a critical role in host defense against Mabc infection. Mabc decreased SIRT3 and upregulated mitochondrial oxidative stress in macrophages. SIRT3 deficiency led to increased bacterial loads, histopathological, and mitochondrial damage, and pathological inflammation during Mabc infection. Administration of scavengers of mitochondrial reactive oxygen species significantly decreased the in vivo Mabc burden and excessive inflammation, and induced SIRT3 expression in infected lungs. Notably, SIRT3 agonist (resveratrol) significantly decreased Mabc growth and attenuated inflammation in mice and zebrafishes, indicating the key role for SIRT3 in metazoan host defense. Collectively, these data strongly suggest that SIRT3 is a host-directed therapeutic target against Mabc infection by controlling mitochondrial homeostasis.

## Introduction

*Mycobacterium abscessus* (Mabc) is an emerging and rapidly growing nontuberculous mycobacterial (NTM) strain that causes numerous human infections, usually associated with lung diseases [1,2]. Mabc is a subspecies of the Mabc complex that includes Mabc, *M. massiliense*, and *M. bolletii*, and has major clinical implications due to antibiotic resistance [2,3]. Mabc often causes pulmonary infection in patients with immune deficiencies, such as cystic fibrosis; however, it can also threaten immunocompetent individuals [4,5]. Mabc is divided into two morphological types, smooth and rough variants, depending on the colony phenotype [6,7]. The Mabc rough (Mabc-R) variant is more virulent and invasive compared to Mabc smooth (Mabc-S) variants. Mabc-R has very minimal glycopeptidolipids (GPLs), which mask the underlying phosphatidyl-myo-inositol mannosides and induce innate immune and inflammatory responses [8]. Although several risk factors (e.g., old age, immune-modulating agents, underlying lung diseases) related to NTM diseases have been reported [5,9], the host factors that are implicated in the pathogenesis during Mabc infection are largely unknown.

Mitochondria are essential intracellular organelle for oxidative energy metabolism, adenosine triphosphate (ATP) production, and calcium homeostasis [10]. In mitochondria, numerous proteins/enzymes associated with respiratory chain reaction, dynamics, and energy metabolism are regulated by lysine acetylation [10]. Sirtuin 3 (SIRT3) is a major deacetylase in the mitochondria [11] and affects mitochondrial functions through nicotinamide adenine dinucleotide (NAD+)-dependent protein deacetylation [12]. Although the function of SIRT3 has been studied in metabolic tissues, accumulating data imply that SIRT3 plays crucial roles in non-metabolic cells and tissues, including lungs and immune cells [10,12––15]. Our recent studies showed that SIRT3 was required for the host’s innate defense against *Mycobacterium tuberculosis* (Mtb) infection. Importantly, SIRT3 contributed to mitochondrial homeostasis and xenophagy activation in macrophages during Mtb infection [16]. However, observations were inconsistent regarding the function of SIRT3 in host defenses against various bacterial and fungal infections, including infections with *Escherichia coli* and *Klebsiella pneumoniae* [15]. In addition, a recent study showed that SIRT3/5 double knockout (KO) mice showed improved resistance to listeria infection [17]. Thus, to date, the putative role of SIRT3 in NTM infection is unknown. Moreover, recent advances and emerging data have revealed SIRT3 modulators, which may ultimately be used as promising drugs for control of numerous human disorders [12]. In this regard, the therapeutic potential of SIRT3-targeted therapy could be validated as a potential drug target for the treatment of Mabc infection *in vivo*.

In this study, we investigated whether mitochondrial homeostasis is critical for host protection against Mabc infection and whether SIRT3-mediated, mitochondria-targeted therapy was beneficial for controlling Mabc infection *in vivo*. Mabc infection led to decreased expression of SIRT3, mitochondrial reactive oxygen species (ROS) generation, and mitochondrial dysfunction in macrophages. We found that SIRT3 deficiency led to increased bacterial loads, mitochondrial ROS and dysfunction, and pathological inflammation against Mabc infection, when compared to wild-type (WT) mice. Compared with Mabc-S, Mabc-R led to more mitochondrial oxidative stress in macrophages. Therefore, we primarily focus on the pathological responses induced by Mabc-R infection throughout the paper. Importantly, the mitochondrial ROS scavenger MIT-001 showed a therapeutic effect against Mabc-R infection through amelioration of pathological inflammation and suppression of mitochondrial ROS. Furthermore, resveratrol (RSV), a SIRT3-activating agent, played a protective role with antimicrobial effects against Mabc-R and – S infection in mice and zebrafish (ZF).

## Material and methods

### Mycobacterial strains and inoculum preparation for infection

The smooth-morphotype Mabc ATCC19977 WT (Mabc-S) and the isogenic rough type (Mabc-R) were used for this study. This genetically identical rough type of Mabc ATCC19977 strain was obtained through continuous anaerobic passage of the WT strain and has previously been used to study Mabc-R virulence factors [18]. The bacteria were incubated at 37ºC using an orbital shaker in Middlebrook 7H9 medium containing 10% oleic albumin dextrose catalase (BD, Franklin Lakes, NJ) until the mid-log phase (OD_600nm_=0.6).

After cultivation, bacterial culture broths were harvested by centrifugation, and the bacterial pellet was washed three times with PBS buffer solution to remove the glycerol and BSA completely. Because these bacteria, particularly Mabc-R, grow into cord-formatted clumps, they were separated into single cells using a tissue homogenizer consisting of a Teflon rod and a glass tube (Wheaton, Millville, NJ, USA) for 2 min at 3000 rpm. After the separation process, the bacterial single-cell suspensions were aliquoted and stored at −70ºC until just before use. Prior to use, the frozen bacterial stocks were thawed in ice and soaked in an ultrasonic bath (As-one, Osaka, Japan) to prevent re-clumping and were used for the infection procedure.

Mabc CIP 104536 ^T^ R and S strains were kindly provided by Dr. Laurent Kremer (Université de Montpellier, Montpellier, France). Mabc CIP 104536 ^T^ R and S morphotypes carrying a pMV262-mWasabi plasmid that allowed the expression of mWasabi were used for the evaluation of bacterial dissemination in ZF [19]. Mid-log phase Mabc strains were harvested and declumped before preparing a frozen stock using a 26-gauge needle and sonication at 40 kHz for 30 s three times (Branson CPX3800, Danbury, CT, USA). Prior to injection, the viable numbers of bacteria were enumerated by plating serially diluted stocks on 7H10 agar. For ZF infection, the inoculum was diluted with phosphate-buffered saline-tween 20 (PBST) 0.05% tween 80 and resuspended in phenol red 0.085% to obtain 130 CFU/mL.

### Mycobacterial infection of mice

Mycobacterial infections in SIRT3 WT and KO mice were performed as previously described [16]. Groups of male 6–8-week-old mice were infected intranasally with Mabc-R (1 x 10^6^ or 10^7^ CFU/mouse) or Mabc-S (1 x 10^7^ CFU/mouse) for 5 or 7 days. At 24 h post-infection, the numbers of bacteria in the lungs of at least three mice were determined to confirm the Mabc-R and Mabc-S inoculum and represent the average inoculum of mice from each group.

### Maintenance of mice and isolation of macrophages

SIRT3 WT and KO mice were kindly provided by Dr. Hyun Seok Kim (Ewha Womans University, Seoul, Korea) and maintained under specific-pathogen-free conditions in Chungnam National University School of Medicine. The mice used in all experiments were 6–8 weeks old and sex-matched. All mice were bred and housed for experiments in accordance with the guidelines of Chungnam National University School of Medicine. Mice experimental protocols were approved by the Institutional Animal Care and Use Committee of Chungnam National University (CNUA-18-0117).

Primary mouse bone marrow-derived macrophages (BMDMs) were isolated and cultured as previously described [16]. BMDMs were isolated and differentiated after culture for 4–5 days in medium containing 25 ng/ml macrophage colony-stimulating factor (R&D Systems, Minneapolis, MIN, USA). Peritoneal macrophages (PMs) were isolated with 2–3 days after injection with 1 ml of PBS supplemented with 3% thioglycollate (Sigma-Aldrich, St. Louis, MO, USA). Prior to isolation, PMs were collected with pre-chilled PBS supplemented with 10% FBS, in mouse abdominal cavity. BMDMs and PMs were cultured with a medium consisting of DMEM supplemented with 10% FBS, and penicillin-streptomycin-amphotericin B at 37°C, 5% CO_2_ atmosphere.

### Antibodies and chemicals

MIT-001 (C25H32N4O4S2; patent no. KR2008-0080519) was provided by MitoImmune Therapeutics, Inc. (Seoul, Korea). Antibodies for SIRT3 (5490S) and ACTB (sc-47778) were purchased from Cell Signaling Technology (Danvers, MA, USA) and Santa Cruz Biotechnology (Dallas, TX, USA), respectively. Antibodies for NDUFA9 (ab14713) and UQCRC2 (ab14745) were purchased from Abcam (Cambridge, UK), for SDHA (5839) and COX4 (4844) were purchased from Cell Signaling Technologies (Danvers, MA, USA), and for ATP5A (459240) was purchased from Thermo Fisher Scientific, respectively. propidium iodide solution (P3566) and mitoSOX^TM^ Red (M36008) were purchased from Invitrogen (Carlsbad, CA, USA). 3-TYP (S8628) was purchased from Selleckchem. Resveratrol was synthesized from 1-ethynyl-3,5-dimethoxybenzene, as described [20].

### Determination of CFUs from Mabc-infected lungs and macrophages

For *in vivo* CFU assays, lungs from the infected mice were harvested at 5 or 7 days, depending on the experimental design. For the measurement of the bacterial burden, the lungs were homogenized in PBST and serial dilutions of the homogenates were plated on duplicate plates of Middlebrook 7H10 agar.

For the quantification of intracellular bacteria from macrophages, CFU assays were performed as previously described [21]. Briefly, Mabc-infected BMDMs were washed with PBS, and fresh medium containing 50 μg/ml gentamicin (Sigma-Aldrich, St. Louis, MO, USA) was added and lysed with 0.3% saponin (Sigma-Aldrich) to release intracellular bacteria. Then, infected lysates were resuspended vigorously, transferred to screw-capped tubes, and sonicated in a preheated 37°C water bath sonicator (Elma, Singen, Germany) for 5 min. Aliquots of the sonicates were diluted fivefold in 7H9 medium and homogenates were plated on duplicate plates of Middlebrook 7H10 agar. Bacterial colonies were counted after 3–5 days of incubation at 37°C.

### Immunohistochemistry (IHC) and PI staining for Mabc-infected lung tissues

Lungs were harvested from mice infected with Mabc for 10 days. Lungs were fixed in 10% formalin and embedded in paraffin wax. For histopathology, lung paraffin sections (4 µm) were cut and stained for hematoxylin and eosin (H&E) as previously described [21]. For analysis of the extent of tissue necrosis, propidium iodide (PI) staining was performed. For analysis of the extent of tissue necrosis, lung paraffin sections (4 µm) were cut and immunostained with propidium iodide solution (P3566; Invitrogen, Carlsbad, CA, USA). After mounting, fluorescence images were acquired using a confocal laser-scanning microscope (LSM 710; Zeiss, CLSM, Jena, Germany), with constant excitation, emission, pinhole, and exposure-time parameters. H&E staining was scanned using an Aperio digital pathology slide scanner (Leica) and imaged using an Aperio ScanScope® CS System. To quantify the inflamed area and necrosis, the MFI of the red threshold was determined using FIJI software.

### RNA extraction and quantitative real-time PCR (qPCR) analysis

RNA extraction and qPCR were performed as previously described [21]. Briefly, total RNA from BMDMs or lung tissues were isolated for using TRIzol reagent (Thermo Fisher Scientific). The cDNA synthesis was performed using Superscript II reverse transcriptase (Invitrogen, 18064). QPCRs were carried out with cDNA, primers, and SYBR Green PCR Kits (Qiagen, 204074) using a Real-time PCR Cycler Rotor-Gene Q 2plex system (Qiagen GmbH, 9001620, Hilden, Germany). The samples were amplified for 50 cycles as follows: 95°C for 5 s and 60°C for 10 s. To analyze qPCR data, we performed relative quantification using the 2^ΔΔ^Ct method with *Gapdh* as an internal control gene; data were expressed as relative fold changes. The primer sequences are shown in **Table S1**.

### Enzyme-linked immunosorbent assay (ELISA)

The concentration of TNF in the lung tissues was measured using commercially available ELISA kit (BD Biosciences, 558534, San Jose, CA, USA). We performed the experiment according to the protocol provided of the manufacturer.

### Western blot analysis

Tissue homogenates were lysed in radioimmunoprecipitation assay (RIPA) buffer (50 mM Tris-HCl, pH 7.5, 150 mM NaCl, 1% NP-40, 0.5% DOC (deoxycholic acid), 0.1% SDS, 1 mM PMSF) (ELPIS Bio, Lexington, MA, USA) supplemented with protease and phosphatase inhibitor cocktail (Roche, Basel, Switzerland). Equal amounts of protein were mixed with 5X SDS sample buffer (ELPIS) and boiled for 5 minutes. Proteins were then separated by sodium dodecyl sulfate-polyacrylamide gel electrophoresis (SDS-PAGE) and transferred to polyvinylidene difluoride (PVDF) membranes (Millipore, Burlington, MA, USA). The membranes were blocked in 5% skim milk in Tris-buffered saline–Tween 20 (TBS–T) for 1 h at room temperature, and were incubated overnight at 4°C with the following specific primary antibodies: anti-SIRT3 (5490, Cell Signaling Technologies), anti-ACTB (sc-47778, Santa Cruz Biotechnology), anti-NDUFA9 (ab14713, Abcam, Cambridge, UK), anti-SDHA (5839, Cell Signaling Technologies), anti-UQCRC2 (ab14745, Abcam), anti-COX4 (4844, Cell Signaling Technologies), anti-ATP5A (459240, Thermo Fisher Scientific). After washing with TBS–T, the membranes were incubated with appropriate horseradish peroxidase-conjugated secondary antibodies (Cell Signaling Technologies) at room temperature for 1 h. Blots were imaged using a chemiluminescence assay kit (Millipore) in a UVitec Alliance mini-chemiluminescence device (BioSPX, Abcoude, Netherlands). Band densities were quantified using ImageJ software, and data were normalized to β-actin loading control.

### Measurement of mitochondrial ROS and immunofluorescence

Mitochondrial ROS were measured as described previously [16]. SIRT3 WT and KO BMDMs were incubated with 3 μM MitoSOX Red Mitochondrial Superoxide Indicator (Invitrogen, M36008). After 20 min, the cells were washed and measured using immunofluorescence analysis. Nuclei were stained by incubation with 4′,6-diamidino-2-phenylindole (Sigma-Aldrich) at the same time. Immunofluorescence images were acquired using a confocal laser-scanning microscope (Zeiss). The mean fluorescence intensity (MFI) level of the mitochondrial ROS was calculated for each sample. Each experiment was carried out in triplicate, and at least 200 cells per well were counted.

### Mitochondrial oxygen consumption rate (OCR) analysis

The XF24 biosensor cartridge was activated with 1 ml of XF24 calibrant solution (Seahorse Bioscience, Billerica, MA) per well for 24 h at 37°C in a non-CO2 incubation system. SIRT3 WT and KO BMDMs infected with Mabc for 18 h were seeded at 2 × 10^4^ cells per well and incubated for 24 h at 37°C. The cell plate was incubated for 1 h at 37°C in a non-CO_2_ incubation system after the addition of 590 µl assay media in each well. ATPase inhibitor oligomycin A (20 µg/mL, Sigma-Aldrich, MO, USA), uncoupler carbonyl cyanide 3-chlorophenylhydrazone (CCCP, 50 µM, Sigma-Aldrich, MO, USA), and mitochondrial complex I inhibitor rotenone (20 μM, Sigma-Aldrich, MO, USA) were sequentially added to each well after measurement of basal OCR. Seahorse Bioscience XF24 analyzer (Seahorse Bioscience, Billerica, MA) was used to measure the oxygen consumption rate of the entire process.

### Transmission electron microscopy (TEM) analysis

SIRT3 WT and KO mouse lung tissues were fixed with 2.5% glutaraldehyde in 0.1 M cacodylate buffer (pH 7.2) containing 0.1% CaCl_2_. After 3 h, the cells were post-fixed with 1% OsO_4_ in 0.1 M sodium cacodylate buffer containing 0.1% CaCl_2_ for 2 h. The tissues were rinsed with cold distilled water and dehydrated slowly using an ethanol series and propylene oxide at 4°C. The tissues were embedded in Embed-812 and cured at 60°C for 30 h. Ultrathin sections (70 nm) were cut with a diamond knife and ULTRACUT UC7 ultramicrotome (Leica) and mounted on formvar-coated copper grids. Sections were stained with 4% uranyl acetate for 7 min and lead citrate for 7 min. TEM-stained sections were scanned using a Bio-High Voltage EM system (JEM-1400 Plus and JEM-1000 BEF; JEOL Ltd., Tokyo, Japan).

### Microinjection of Mabc into ZF through caudal vein infection for drug efficacy

All ZF studies were conducted under the approval of the ethics committee concerning animal research at Gyeongsang National University (GNU-190325-E0014). The microinjections of Mabc into ZF were conducted as previously described [19]. Briefly, dechorionated and anesthetized ZF were injected with 3 nL of Mabc (~400 CFU) into the caudal vein using a Nanoject III Programmable Nanoliter Injector (Drummond Scientific, Broomall, PA, USA). After injection, the infected ZF were transferred into 96-well plates (two embryos/well) and grown in fish water supplemented with 1 g/L methylene blue at 28.5°C. For drug efficacy evaluation, three concentrations of RSV (1, 5, and 10 µM) were tested in the Mabc-infected ZF by direct addition to fish water for 5 days. Mabc-infected ZF treated with DMSO (1%) were used as a negative control and clarithromycin(CLA)-treated ZF were used as a positive control. The fish water including the drugs was renewed once every day. The *in vivo* drug efficacy was determined for each concentration by Mabc-mWasabi dissemination, bacterial burdens in ZF and kinetics of ZF survival. To evaluate the drug efficacy with Mabc-mWasabi dissemination in ZF, a SteREO Lumar.V12 stereomicroscope (Zeiss) with fluorescence optics was used after treatment with RSV and CLA. For the survival rate after treatment compounds, dead embryos were recorded on a daily basis for 13 days and a Kaplan-Meier curve was used as previously described [19]. For the quantification of the bacterial burden, three infected ZF (5 dpi) per group were collected and individually homogenized in 2% Triton X-100–PBST using a hand-held homogenizer (D1000; Benchmark Scientific, Sayreville, NJ, USA). Several 10-fold dilutions of the suspension in PBST were plated on 7H10 containing BBL MGIT PANTA (polymyxin B, amphotericin B, nalidixic acid, trimethoprim, and azlocillin; Becton Dickinson, Franklin Lakes, NJ, USA).

### Statistical analysis

Statistical analysis was performed in Prism (GraphPad Software, v5.01, 2007). Data are presented as means ± SEM (standard error of the mean). Data were analyzed using a two-tailed Student’s *t*-test or non-parametric tests. In non-parametric tests, two conditions were compared using Mann-Whitney U-Test and three or more conditions using one-way ANOVA with Dunn’s multiple comparison test where appropriate. Specific *p* values are detailed in the figure legends. For the ZF survival study, Kaplan-Meier survival curves were generated and analyzed by a Gehan-Breslow-Wilcoxon test.

## Results

### SIRT3 is required for host defense against Mabc infection

**Figure 1.**
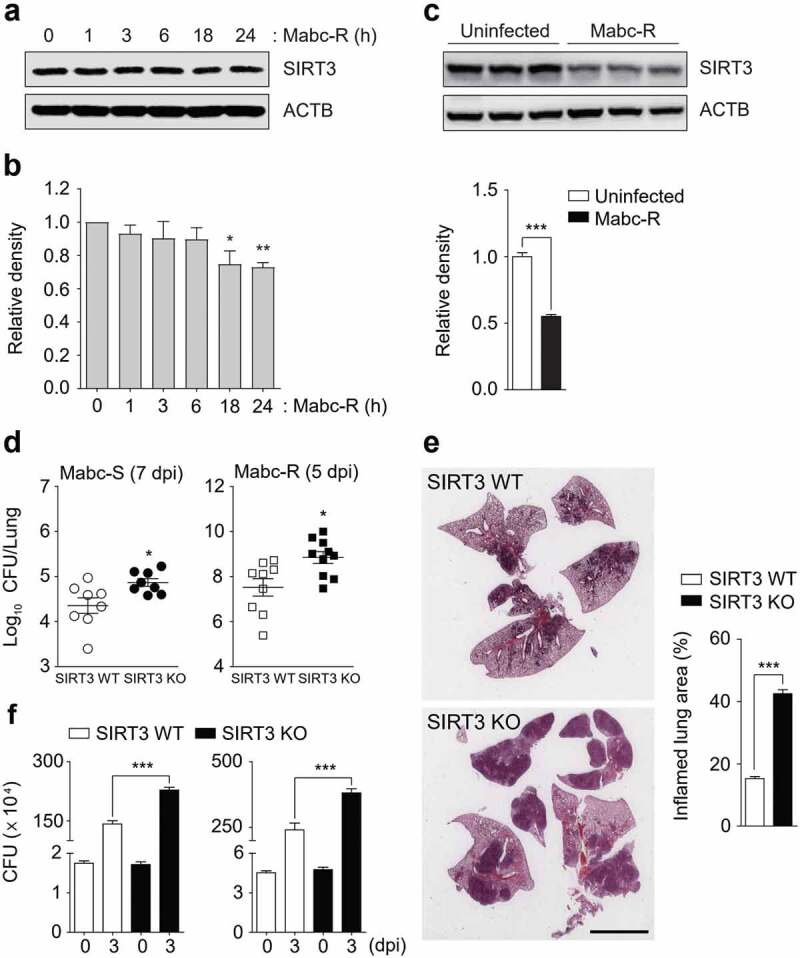
SIRT3 is essential for host defense against mycobacterial infection *in vivo* and *in vitro*. (a and b) WT BMDMs were infected with Mabc-R (MOI = 3) at the indicated times. Actin protein levels were evaluated by immunoblotting as an internal control. (b) Quantification of results on (a). (c) Western blot analysis of the lung tissues from SIRT3 WT and KO mice left uninfected or infected intranasally with Mabc-R (1 × 10^7^ CFU) for 3 days. Quantifiation of results on top. (d) SIRT3 WT and KO mice were infected intranasally with various CFUs of Mabc-R (1 × 10^7^ CFU) or Mabc-S (1 × 10^7^ CFU) and monitored at 5 or 7 days post-infection (dpi). Data are shown as log pulmonary CFU. (e) Lung histopathology by H&E staining of SIRT3 WT and KO mice infected with Mabc-R for 5 days. Right, Quantification of results on left. Scale bar, 5 mm. (f) Intracellular survival of Mabc-S assessed by a CFU assay. SIRT3 WT and KO BMDMs were infected with Mabc-S (MOI = 1, for left; MOI = 3, for right) for 4 h, and then lysed to determine intracellular bacterial loads at 0 and 3 dpi. *P < 0.05, **P < 0.01, ***P < 0.001. Non-parametric test (b and d); Student’s *t*-test (c below and e right); One-way ANOVA (f). Data represent three independent experiments (a, c top, and e left), and values represent means (± SEM) from three or four independent experiments performed in triplicate (b, c bottom, e right, and f).

Given our previous findings of SIRT3 during Mtb infection [16], we first investigated whether Mabc infection decreased the levels of SIRT3 in macrophages and *in vivo*. We found that Mabc-R infection significantly decreased expression of SIRT3 in macrophages in a time-dependent manner (Figure 1(a,b)). When SIRT3 WT mice were intranasally infected with Mabc-R, SIRT3 protein levels were significantly reduced (~ 2 fold) at 1 day post-infection (dpi) in the lung tissues from mice (Figure 1(c)). Next, we examined the role of SIRT3 in the antimicrobial response *in vivo* by using SIRT3 WT and SIRT3 KO mice. SIRT3 WT and SIRT3 KO mice were intranasally infected with Mabc-R or Mabc-S. *In vivo* bacterial loads in the lungs were significantly higher in SIRT3 KO mice than in SIRT3 WT mice after infection with Mabc-R or Mabc-S (Figure 1(d)). There was no significant difference in the inoculum dose of Mabc between the SIRT3 WT and KO mice (Figure S1).

Additionally, the SIRT3 KO mice had enhanced lung pathology at 5 dpi with Mabc-R (i.e., more granulomatous and inflammatory lesions in the lungs) than did the SIRT3 WT mice (Figure 1(e)). The intracellular survival assays revealed that the SIRT3 KO BMDMs had significantly higher intracellular Mabc-S (multiplicity of infection [MOI] = 1 and 3) compared to the SIRT3 WT BMDMs (Figure 1(f)). Since Mabc-R infection significantly induced cell death at a MOI of 1 (2 dpi; data not shown), we did not perform an intracellular survival assay using Mabc-R. Collectively, these data indicate that SIRT3 contributes to antimicrobial responses against Mabc-R and Mabc-S infection.

### SIRT3 is required for the amelioration of pathologic inflammation and controlling mitochondrial damage during Mabc-R infection

**Figure 2.**
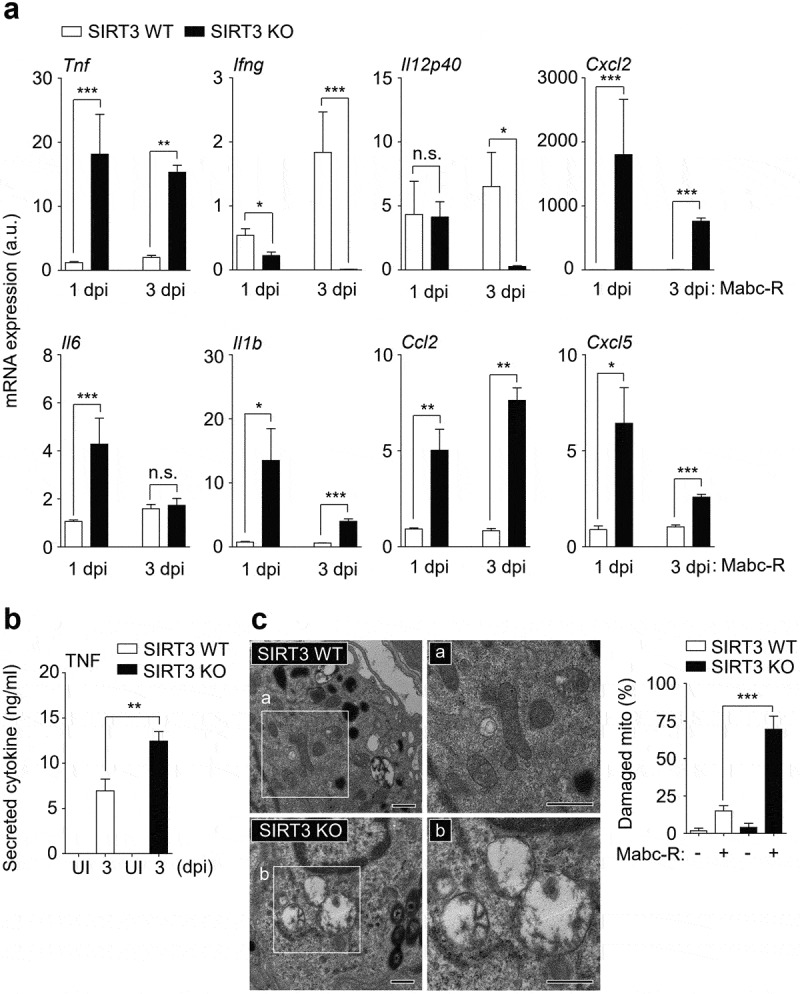
SIRT3 is required to control pathological inflammation and mitochondrial damage during Mabc-R infection. (a and b) SIRT3 WT and KO mice (*n* = 8 each group) were infected intranasally with Mabc-R (1 × 10^7^ CFU) and monitored at 1 and 3 dpi. (a) Lung tissues were subjected to quantitative real-time PCR analysis for the measurement of mRNA expression of various cytokines/chemokines. (b) The supernatants from lung lysates were subjected to ELISA analysis of TNF (at 3 dpi). (c) SIRT3 WT and KO mice (*n* = 3 each group) were infected intranasally with Mabc-R (1 × 10^7^ CFU) and monitored at 5 dpi. The lung tissues were harvested and then subjected to TEM analysis (left). Mitochondria with complete cristae are shown in a; swollen mitochondria with vacuolation in the cristae are shown in b. Right, Quantitative analysis of at least 8 EM images in the lung tissues from SIRT3 WT and KO mice infected intranasally with Mabc-R (1 × 10^7^ CFU; *n* = 3 each group). The ratio of damaged mitochondria in total mitochondria was calculated quantitatively. Scale bars, 500 nm. *P < 0.05, **P < 0.01, ***P < 0.001, n.s., not significant compared with SIRT3 WT conditions (a). Non-parametric test (a, b, and c right). Data represent three independent experiments (c left), and values represent means (± SEM) from three or four independent experiments performed in triplicate (a and b).

Since the Mabc-R variant is more virulent and active in the induction of inflammation than Mabc-S [8], we next compared the lung inflammatory responses between SIRT3 WT and SIRT3 KO mice using the Mabc-R strain. To examine this, we collected lung tissues at 1 and 3 dpi, and performed qRT-PCR analysis for the mRNA levels of proinflammatory cytokines/chemokines. As shown in Figure 2(a), the mRNA expression levels of a variety of proinflammatory cytokines/chemokines (*Tnf, Il1b, Il6, Cxcl2, Ccl2*, and *Cxcl5*) were significantly higher in lung tissues from the SIRT3 KO mice than in those from SIRT3 WT mice at 1 and 3 dpi. The protein level of TNF was also upregulated in the lung tissues from SIRT3 KO mice than in those from SIRT3 WT mice (Figure 2(b); at 3 dpi). However, both *Ifng* and *Il12p40* mRNA expression levels were markedly depressed in the lungs from SIRT3 KO mice relative to those from SIRT3 WT mice (Figure 2(a)).

It is well established that SIRT3 is essential in mitochondrial homeostasis and functions to protect various cells and tissues from stress-induced cell death [22––25]. In addition, we reported that SIRT3 was required for the maintenance of mitochondrial homeostasis during Mtb infection [16]. When we next performed ultrastructural analysis between SIRT3 WT and KO lungs after infection, the TEM data showed that SIRT3-deficient lungs had a marked accumulation of damaged mitochondria, as represented by swollen and disrupted cristae, when compared with SIRT3 WT mice at 5 dpi of Mabc-R infection (Figure 2(c)). However, there was no significant difference in mitochondrial morphologies between SIRT3 WT and KO lungs prior to infection (Figure 2(c) right and S2). These data imply that SIRT3 deficiency results in a marked increase in mitochondrial damage and excessive inflammatory responses during Mabc-R infection.

### SIRT3 deficiency leads to increased inflammatory responses and mitochondrial oxidative stress in macrophages during Mabc-R infection

**Figure 3.**
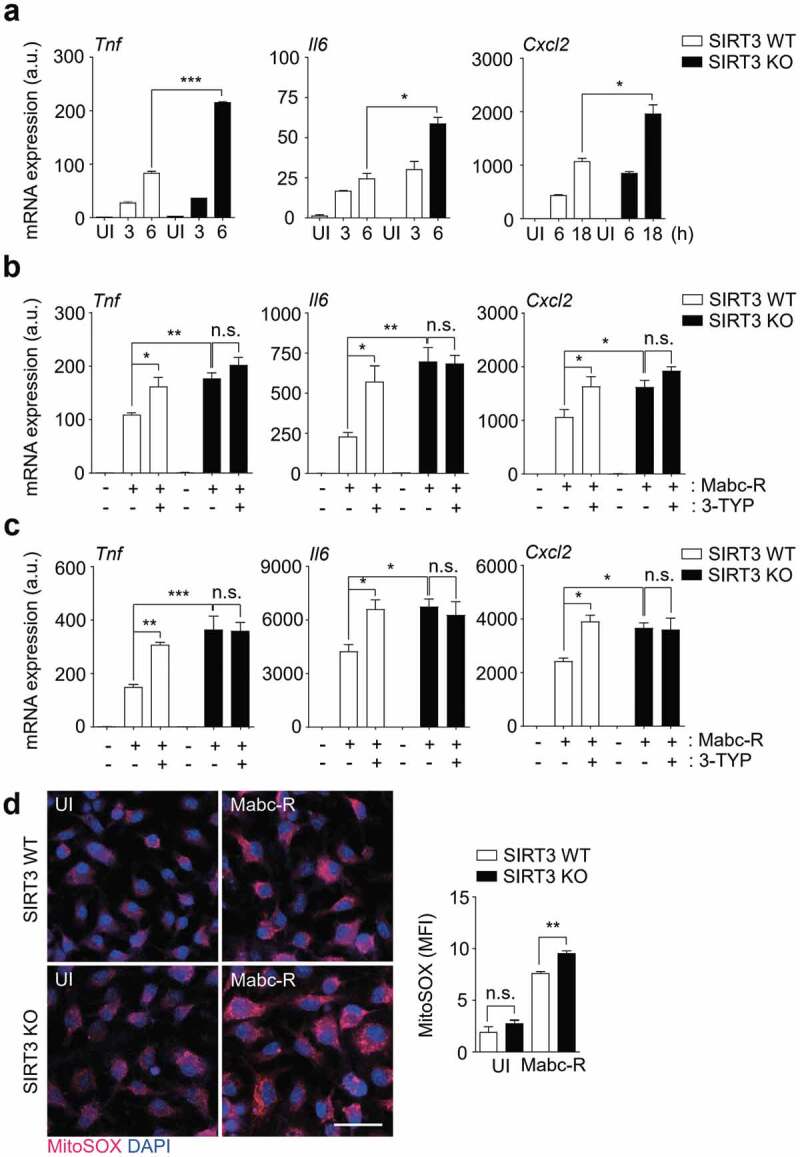
SIRT3 is essential for the amelioration of proinflammatory cytokine expression and controlling mitochondrial ROS production in BMDMs during Mabc-R infection. (a) BMDMs from SIRT3 WT and KO mice were infected with Mabc-R (MOI = 3) and incubated for 3, 6, or 18 h. (b and c) BMDMs (b) and PMs (c) were prepared from SIRT3 WT and KO mice, and were infected with Mabc-R (MOI = 3) in the presence or absence of 3-TYP (50 µM) for 3 h and quantitative real-time PCR analysis for *Tnf, Il6*, and *Cxcl2* was performed. (d) SIRT3 WT and KO BMDMs were infected with Mabc-R (MOI = 3) for 2 h and subjected to MitoSOX Red staining (representative images are shown in the left panel; quantitative analysis is shown in the right panel). Scale bar, 50 μm. *P < 0.05, **P < 0.01, ***P < 0.001. U, uninfected. One-way ANOVA (a-d). Data are representative of three independent experiments (d left), and values represent means (± SEM) from three or four independent experiments performed in triplicate (a-c, d right).

We next compared the mRNA expression of inflammatory cytokines in BMDMs from SIRT3 WT and SIRT3 KO mice after Mabc-R infection. Mabc-R-mediated mRNA generation of *Tnf, Il6*, and *Cxcl2* was significantly increased in BMDMs from SIRT3 KO mice compared to SIRT3 WT mice after infection in a time-dependent manner (Figure 3(a)). The protein levels of TNF were also increased in SIRT3 KO BMDMs compared to SIRT3 WT BMDMs after Mabc-R infection (data not shown). Similarly, we performed qRT-PCR analysis of proinflammatory cytokines (*Tnf, Il6*, and *Cxcl2*) in both SIRT3 WT and SIRT3 KO BMDMs or PMs, which were pretreated with 3-TYP prior to Mabc-R infection. As shown in Figure 3(b,c), we found that pretreatment of SIRT3 WT BMDMs or PMs with 3-TYP significantly increased the expression of the mRNAs of *Tnf, Il6*, and *Cxcl2* in response to Mabc-R. When compared with SIRT3 WT BMDMs or PMs, SIRT3 KO BMDMs or PMs showed a significantly increased mRNA levels of *Tnf, Il6*, and *Cxcl2* after infection with Mabc-R. However, 3-TYP pretreatment had no significant effect in mRNA expression of those proinflammatory cytokines in SIRT3 KO BMDMs or PMs, after infection with Mabc-R (Figure 3(b,c)). These data strongly suggest that SIRT3 inhibition increases the expression levels of *Tnf, Il6*, and *Cxcl2* in macrophages after infection with Mabc-R.

Previous studies demonstrated that mitochondrial ROS generation is modulated by SIRT3 in a variety of cells [26––29]. Thus, the mitochondrial redox status was compared between SIRT3 WT and SIRT3 KO BMDMs using MitoSOX Red, a highly selective fluorescent probe for the detection of mitochondrial O_2_^−^ [30]. We first examined whether Mabc-R generated more mitochondrial ROS in WT BMDMs than Mabc-S. We infected BMDMs with either Mabc-R or Mabc-S and found that Mabc-R led to more mitochondrial ROS production at 2 h after infection than Mabc-S did (Figure S3). In addition, we found that mitochondrial O_2_^−^ generation was significantly increased in SIRT3 KO BMDMs, when compared with SIRT3 WT BMDMs, after Mabc-R infection (Figure 3(d)). These data imply that SIRT3 deficiency exaggerated oxidative stress and upregulated proinflammatory cytokine expression in macrophages during Mabc-R infection.

### SIRT3 is essential for the maintenance of OXPHOS function and blockade of exaggerated cell death during Mabc-R infection

**Figure 4.**
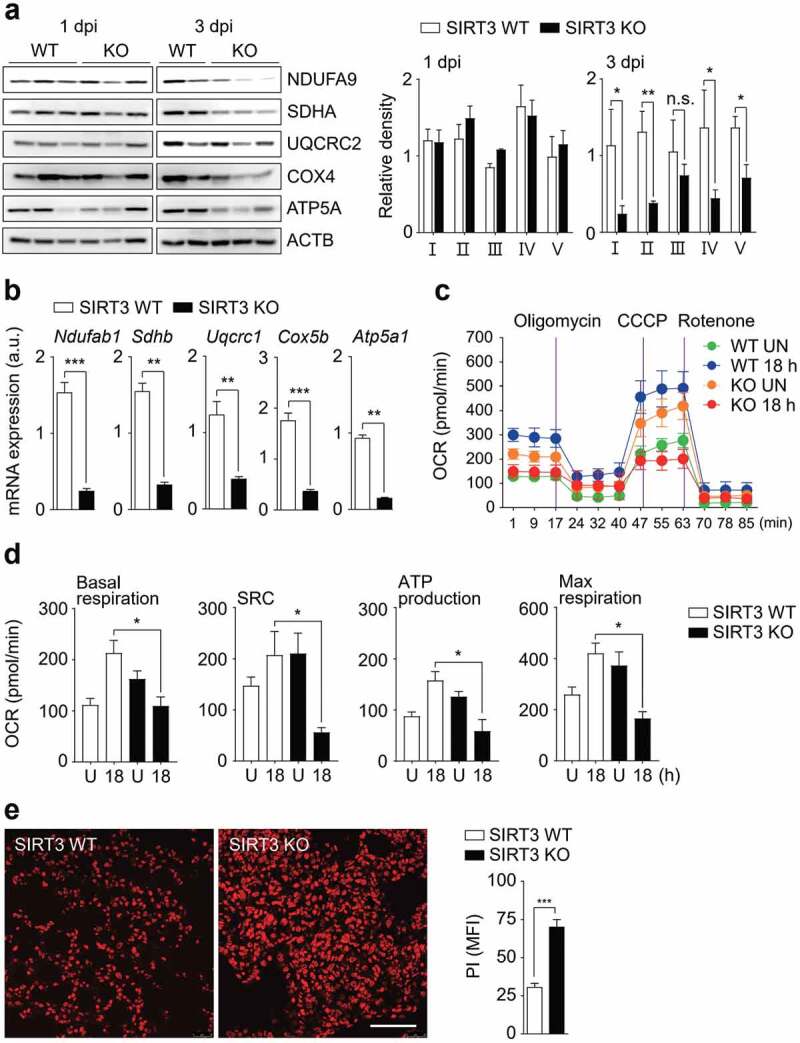
SIRT3 is required for mitochondrial OXPHOS function and attenuation of cell death during Mabc-R infection. (a and b) SIRT3 WT and KO mice (*n* = 8 each group) were infected intranasally with Mabc-R (1 × 10^7^ CFU) and monitored at 1 and 3 dpi. Lung tissues were collected and subjected to (a) Western blot analysis for measurement of OXPHOS protein expression (Left, representative images; right, quantitative analysis), and (b) qRT-PCR analysis. (c) Oxygen consumption rate (OCR) analysis of SIRT3 WT and KO BMDMs untreated or treated with Mabc-R for 18 h. (d) Quantitative analysis of basal respiration, spare respiratory capacity (SRC), ATP production, and maximal respiration analysis from (c). (e) PI staining after infection (Left, representative images; right, quantitative analysis). Scale bar, 300 μm. *P < 0.05, **P < 0.01, ***P < 0.001. n.s., not significant. Paired *t*-test (a right); non-parametric test (b and e right); One-way ANOVA (d). Data are representative of three independent experiments (a left, c, and e left), and values represent means (± SEM) from three or four independent experiments performed in triplicate (a right, b, d, and e right).

The mitochondrial oxidative phosphorylation (OXPHOS) system is essential for energy production and cellular homeostasis [31]. We further determined the major mitochondrial protein levels in SIRT3 WT and KO lungs during Mabc-R infection. We found that the protein expression levels of OXPHOS were dramatically suppressed in the SIRT3 KO lungs, when compared with those from SIRT3 WT lungs, at 3 dpi of Mabc-R infection (Figure 4(a)). We then assessed a differential expression profile of mitochondrial OXPHOS genes in the lungs from SIRT3 WT and KO mice infected with Mabc-R (Figure 4(b)). Notably, the gene expression of mitochondrial OXPHOS was significantly depressed in the lungs from SIRT3 KO mice, when compared with those from SIRT3 WT mice, at 5 dpi of Mabc-R infection (Figure 4(b)). We further analyzed bioenergetic signatures in BMDMs from SIRT3 WT and SIRT3 KO mice by measuring OCR using the Seahorse XF24 analyzer (Figure 4(c,d). In SIRT3 KO BMDMs, basal respiration, mitochondrial spare respiratory capacity, ATP production, and maximal respiration were significantly decreased, compared to those in SIRT3 WT BMDMs, after Mabc-R infection (Figure 4(c,d)). Combined with the data of mRNA and protein expression of mitochondrial respiratory chain complexes, these data strongly suggest that SIRT3 is required for the maintenance of mitochondrial respiration during Mabc-R infection.

Given the findings that SIRT3 deficiency results in increased mitochondrial defects and ROS production, but depressed OXPHOS activity, we next examined whether cell death was upregulated in the lungs from SIRT3 KO mice relative to those from SIRT3 WT mice. We previously showed that Mabc-R variants induced greater cell death in RAW264.7 cells than did the smooth strain [18]. Thus, we elucidated whether Mabc-R infection led to activation of PI-positive cell death in infected lungs from mice. As shown in Figure 4(e), PI staining of lung tissues showed that cell death was markedly increased in the lung tissues from SIRT3 KO mice compared with those from SIRT3 WT mice after Mabc-R infection. Together, these data imply that mitochondrial OXPHOS function was dramatically downregulated, but host cell death was markedly upregulated, in the SIRT3 KO lungs during Mabc-R infection.

### Blockade of excess mitochondrial ROS enhances the antimicrobial response and ameliorates pathological inflammation during Mabc-R infection

**Figure 5.**
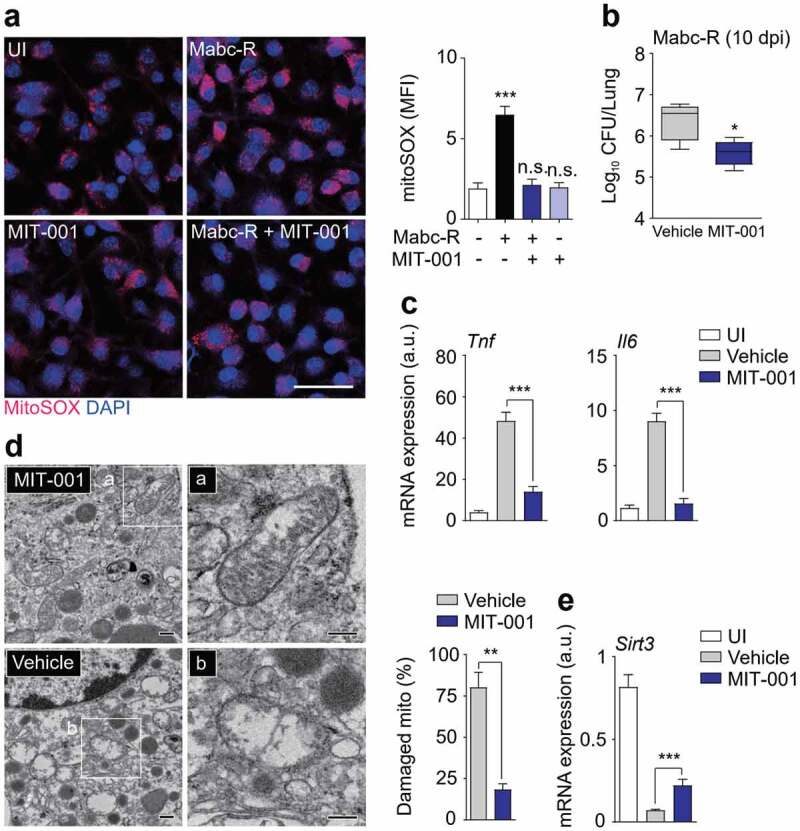
Administration of MIT-001 in mice led to a protective effect against Mabc-R infection. (a) WT BMDMs were infected with Mabc-R (MOI = 5) for 2 h in the presence or absence of MIT-001 (20 µM) and subjected to MitoSOX Red staining (Left, representative images; right, quantitative analysis). Scale bar, 50 μm. (b-e) WT mice (*n* = 5 each group) were left uninfected or infected intranasally with Mabc-R (1 × 10^7^ CFU), prior to treatment with or without MIT-001 (30 mg/kg) and monitored at 10 days post-infection (dpi). Lung tissues were subjected to a (b) pulmonary CFU assay, (c) qRT-PCR analysis for cytokines/chemokines, (d) TEM analysis. Mitochondria with complete cristae are shown in a; swollen mitochondria with vacuolation in the cristae are shown in b. Right, Quantitative analysis of at least 8 EM images in the lung tissues from each group of mice infected intranasally with Mabc-R (1 × 10^7^ CFU; *n* = 3 each group). The ratio of damaged mitochondria in total mitochondria was calculated quantitatively. Scale bars, 200 nm. (e) qRT-PCR analysis for *Sirt3* mRNA expression. *P < 0.05, **P < 0.01, ***P < 0.001, n.s., not significant. One-way ANOVA (a right) or non-parametric test (b, c, d right and e). Data represent three independent experiments (a left and d), and values represent means (± SEM) from three or four independent experiments performed in triplicate (a right, b, c, and e).

The mitochondrial ROS scavenger MIT-001 (previously, NecroX-7) abrogates mitochondrial ROS, calcium, and reactive nitrogen species [32,33]. As expected, treatment of BMDMs with MIT-001 markedly suppressed generation of mitochondrial ROS in response to Mabc-R infection (Figure 5(a)). We next determined whether inhibition of mitochondrial ROS generation enhanced antimicrobial effects *in vivo*. Mice were intranasally infected with Mabc-R, treated with MIT-001 (at 1, 3, 5, 7, and 9 dpi), and sacrificed at 10 dpi for determination of *in vivo* CFUs. MIT-001 significantly inhibited the *in vivo* bacterial loads in the lungs of infected mice (Figure 5(b)), implying that inhibition of excessive mitochondrial ROS is beneficial to control bacterial replication against Mabc infection.

We next examined the effects of MIT-001 on pathological inflammatory responses during Mabc-R infection *in vivo*. The mRNA expression of *Tnf* and *Il6* was dramatically increased in the mouse lungs after Mabc-R infection (Figure 5(c)). Administration of MIT-001 markedly ameliorated the expression of numerous inflammatory cytokines/chemokines, including *Tnf, Il1b, Il6, Cxcl2*, and *Cxcl5*, in the lung tissues from mice infected with Mabc-R (Figure 5c and S4a). In addition, TNF secretion was significantly down-regulated in the lung tissues from Mabc-R-infected mice, by treatment with MIT-001 (Figure S4b).

To analyze the mitochondrial damage *in vivo* further, we performed TEM analysis of lung tissues from Mabc-R-infected mice with or without MIT-001 treatment. After treatment with MIT-001, there were considerably fewer damaged mitochondria with disrupted cristae in the alveolar cells of Mabc-R-infected lung tissues of mice than in control mice (Figure 5(d)). In addition, *Sirt3* mRNA level was markedly inhibited in the mouse lungs after infection with Mabc-R, and significantly recovered after treatment with MIT-001 (Figure 5(e)). Together, these data demonstrated that *in vivo* bacterial growth and pathological inflammation were significantly reduced by MIT-001 treatment, implying that controlling mitochondrial ROS is essential for promoting host defense during Mabc-R infection.

### RSV treatment enhances the antimicrobial response and ameliorates pathologic inflammation during Mabc infection

**Figure 6.**
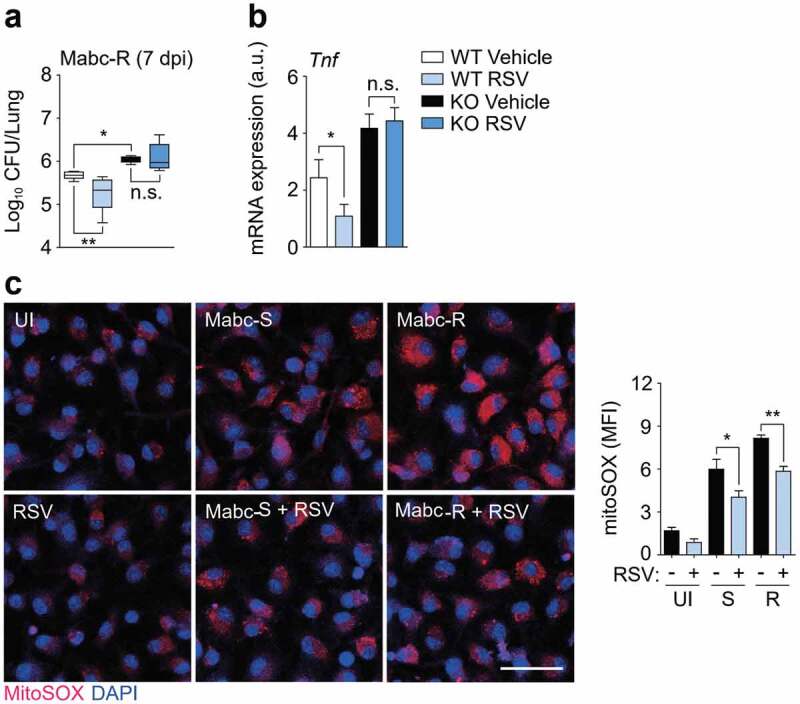
SIRT3 activation by RSV enhances antimicrobial responses and ameliorates mitochondrial oxidative stress during Mabc-R infection. (a and b) SIRT3 WT and KO mice (*n* = 4 each group) were infected with Mabc-R (1 × 10^6^ CFU), followed by treatment with or without RSV (50 mg/kg), and monitored at 7 dpi. (a) Data are shown as log pulmonary CFUs. (b) The lung tissues of the mice were subjected to qRT-PCR analysis. (c) MitoSOX Red staining for Mabc-infected WT BMDMs in the presence or absence of RSV (20 µM). Left, representative images; right, quantitative analysis. Scale bar, 50 μm. *P < 0.05, **P < 0.01, ***P < 0.001. Non-parametric test (a and b; c right); Data represent three independent experiments (c left), and values represent means (± SEM) from three or four independent experiments performed in triplicate (a, b, and c right).

RSV is an activator of SIRT1 and SIRT3, particularly in metabolic and cardiac pathologies [34–36]. We first determined whether treatment with RSV increased antimicrobial effects *in vivo*. SIRT3 WT and KO mice were intranasally infected with Mabc-R and then treated with RSV for 4 days prior to the determination of *in vivo* CFU. RSV significantly inhibited the *in vivo* bacterial loads in the lungs of SIRT3 WT mice, whereas it did not in SIRT3 KO mice (Figure 6(a)). Consistent with this, histopathological damage was markedly ameliorated in the lungs by the administration of RSV (data not shown). Additionally, administration of RSV to the Mabc-S-infected mice significantly attenuated bacterial growth in the lung tissues from the infected WT mice (Figure S5a).

We next examined the effects of RSV on pathological inflammatory responses during Mabc infection *in vivo*. Administration of RSV ameliorated the expression of *Tnf* in the lung tissues from mice infected with Mabc-R in a SIRT3-dependent manner (Figure 6(b)). Similarly, RSV administration significantly attenuated the Mabc-S-induced expression of TNF in the lungs of infected mice (Figure S5b). Moreover, RSV treatment of WT BMDMs led to a marked inhibition of mitochondrial ROS induced by Mabc-S or Mabc-R infection (Figure 6(c)). Together, these data demonstrate that *in vivo* Mabc growth and pathologic inflammation were significantly reduced by RSV treatment by controlling mitochondrial ROS generation.

### In vivo *RSV efﬁcacy assessment using ZF embryos*

**Figure 7.**
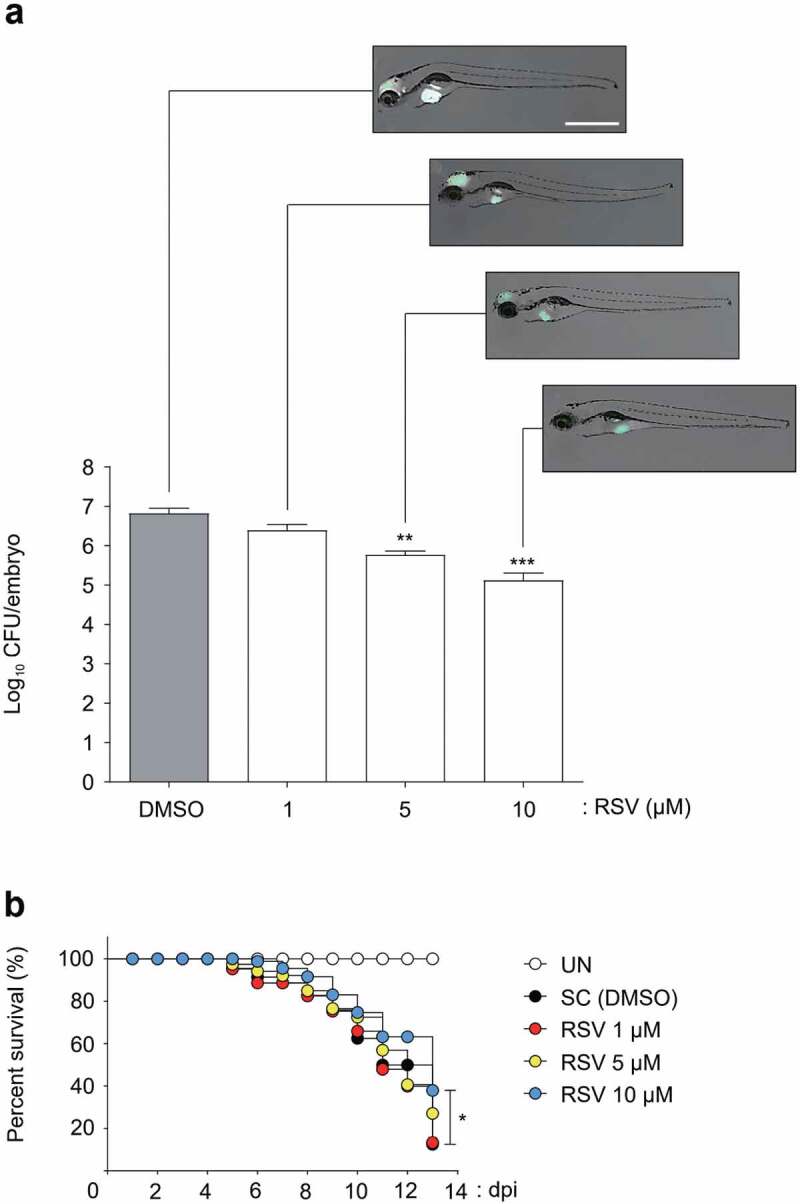
Efficacy of the RSV in Mabc-R infected ZF. (a) The panels show representative dissemination of Mabc-R-mWasabi in ZF. The fluorescent bacterial dissemination and bacterial burden after treatment with different doses of RSV (1, 5, or 10 µM) were assessed. Statistical significance was determined by ANOVA using Tukey’s multiple comparison test. **P < 0.01, ***P < 0.001. Scale bar, 0.5 mm. (b) Survival curve of embryos infected with Mabc-R. The embryos were infected with Mabc-R, followed by treatment with RSV or solvent control (S. C.; DMSO, 1%). As a positive control, ZF without infection was used. RSV, Resveratrol; UNT, untreated. *P < 0.05.

The *in vivo* RSV efficacy was tested in a ZF model of infection with Mabc-R. To determine a suitable concentration of RSV, a maximum tolerated dose (MTD) evaluation was carried out. Non-Mabc infected ZF were treated with several different concentrations (1, 5, 10, 20 and 40 μM) of RSV, and their survival was monitored for 13 days. RSV showed toxicity in the ZF at 20 and 40 μM (Figure S6), and all fish died within 7 days at the concentrations. Comparatively, ZF treated with lower concentrations of RSV such as 1, 5, or 10 μM did not show reduced survival until 13 days after treatment. Based on this compound toxicity result, we injected ~400 CFU of Mabc-R into ZF and treated them with 1, 5, or 10 μM of RSV to evaluate the RSV *in vivo* efficacy in the ZF model. The dissemination of Mabc-R expressing mWasabi in ZF was observed under a fluorescence microscope. The infected Mabc-R-mWasabi was significantly disseminated in the DMSO-treated control in the ZF yolk and head at 5 dpi (Figure 7(a)). However, treatment of Mabc-R-mWasabi infected ZF with 1 or 5 μM of RSV produced a gradual decrease in the degree of the fluorescence signal in a dose-dependent manner. Furthermore, significant green fluorescence reduction in ZF was observed when treated with 10 μM (Figure 7(a)). Only a limited green fluorescence signal was observed in the yolk region. A similar decrease in fluorescent bacterial dissemination by RSV was also observed in ZF infected with the *Mabc*-mWasabi S morphotype (Figure S7a).

Furthermore, this limited bacterial dissemination by RSV was validated by determining the CFUs to confirm the reduction in the bacterial burden inside ZF infected with Mabc-R. After treatment of Mabc-R-infected ZF with different concentrations of RSV (1, 5, or 10 μM), fish were individually homogenized and their viable CFUs were determined. The DMSO-treated negative control showed a maximum live bacterial burden at 5 dpi (Figure 7(a)). However, RSV significantly reduced the persistence of viable bacteria within ZF in a dose-dependent manner. After 5 days of treatment, a reduction in the 1.1 log CFU/embryo was achieved when ZF were treated with 5  μM of RSV. Furthermore, RSV also caused a 1.7 log reduction at a concentration of 10  μM. Again, a similar result of RSV treatment was obtained in ZF infected with the Mabc-S morphotype (Figure S7a).

Next, to measure the survival percentage, a Kaplan–Meier survival curve was plotted. Nearly 100% of non-infected ZF survived until day 13 (Figure 7(b)). By contrast, 90% of Mabc-R-infected ZF that did not receive the drug died by day 13. However, fish treated with 10 μM of RSV showed extended lifespans. Indeed, 38% of Mabc-R morphotype-infected ZF survived up to 13 days in the presence of 10 μM of RSV. This indicates that RSV is an efficient compound for treating Mabc-R infected ZF. Again, RSV also showed potent properties similar to the S morphotype (Figure S7b). These results demonstrate that RSV was an effective compound for treating Mabc in the ZF *in vivo* model.

## Discussion

SIRT3 is fundamentally important for the maintenance of mitochondrial homeostasis under various stress conditions, including genotoxic and metabolic stresses [37]. Although we recently showed that SIRT3 is required for host protection against Mtb infection [16], it is unknown whether SIRT3 activation or deficiency affect the host’s innate defenses during NTM infection. Here, we present evidence that SIRT3 plays a protective role in antimicrobial defense against Mabc infections and that SIRT3-targeted RSV promotes antimicrobial defenses and amelioration of the pathologic inflammation that characterizes Mabc infection.

Mabc-R exhibits various important features, including low levels of GPL but increased virulence and inflammation in mouse-infection models [38,39]. Mabc-R uses invasive patterns to escape from innate host defenses [39]. Indeed, Mabc GPL is essential for the environmental colonization and formation of biofilms [40] and masks the underlying cell-wall phosphatidyl-myo-inositol mannosides that block Mabc interaction with innate receptor toll-like receptor (TLR) 2 [41,42]. In addition, either temperature- or chemical-dependent loss of GPL from R and S variants results in innate immune activation and inflammatory cytokine secretion in macrophages [41]. In this study, we found that Mabc-R greatly upregulated mitochondrial ROS and inflammatory responses in macrophages, compared to Mabc-S. These data partly corroborate previous findings that GPL targeted mitochondria and inhibited macrophage apoptosis induced by the Mabc-R strain through suppression of ROS generation [18].

We aimed to determine whether Mabc-R-mediated defects in mitochondrial morphology and functions were aggravated in SIRT3-deficient macrophages and lungs from mice. We found that, in SIRT3-deficient lungs, Mabc-R significantly increased mitochondrial oxidative stress and down-regulated expression of genes involved in mitochondrial functions (e.g., mitochondrial membrane potential and OXPHOS proteins and genes). These data are partly in agreement with our previous findings in Mtb-infected lungs comparing mitochondrial damage and ROS generation between SIRT3 WT and KO mice [16]. In addition, we found that mitochondrial respiration and membrane potential were markedly downregulated in SIRT3 KO BMDMs after Mabc infection (see Figure 4; data not shown). Nevertheless, we cannot exclude the SIRT3-induced maintenance of mitochondrial function in T cells in terms of protective immunity against Mabc infection. Future studies are warranted to clarify the roles of SIRT3 in T cell activities during Mabc infection. Together, these data imply that defective mitochondrial function plays a detrimental role in host defense against Mabc infection.

*In vivo* administration of the mitochondrial ROS scavenger MIT-001 inhibited *in vivo* bacterial loads and histopathological damage through regulation of excessive production of mitochondrial ROS in macrophages infected with Mabc. The same indole derivative, NecroX-7, was previously reported to improve hepatic steatosis and fibrosis through regulation of cellular ROS and reactive nitrogen species [32]. Another study showed that NecroX-7 exhibited protective ability in a model of myocardial ischemia-reperfusion injury through prevention of Ca^2+^ influx and suppression of mitochondrial permeability transition pore formation [33]. These effects were reported to be even greater than those induced by cyclosporine A [33]. Additionally, treatment of TNF-high ZF with Necrox-5 counteracted the increase of mycobacterial burdens through modulation of excessive mitochondrial ROS [43]. Indeed, previous studies have indicated that mitochondrial ROS generation contributes to the upregulation of pathological inflammatory responses during infection [44]. In addition, dysfunctional mitochondria and oxidative stress are associated with defective microbicidal activities in human monocyte-derived macrophages [45,46]. Together with previous studies, these results strongly imply that dysfunctional mitochondria and ROS generation contribute to the defective innate immune responses to Mabc infection.

Inflammatory responses are a double-edged sword during infection and immunity. TNF signaling was reported to be essential for protective Mabc granuloma formation through IL-8-dependent neutrophil trafficking since TNFR1 deficiency led to increased lethality in ZF Mabc-R infection models [47]. However, excessive TNF production, combined with mitochondrial ROS generation, is deleterious in controlling mycobacteria, the release of bacteria into the extracellular milieu, pathological responses, and necrosis in ZF during mycobacterial infection [43,48]. Notably, our data show increased necroptosis in SIRT3-deficient lungs after Mabc-R infection. A recent study reported that mitochondrial ROS lead to production of lysosomal ceramide to activate the cytosolic protein BAX, ultimately inducing cyclophilin-D-mediated programmed necrosis during *M. marinum* or Mtb infection [48]. However, these data are important in providing evidence that Mabc-R infection results in induction of necrotic cell death, as stained by PI *in vivo*. In addition, previous studies revealed that the virulence of Mabc-R is, at least partly, mediated by increased apoptosis and ROS, and was ameliorated by the cell wall polar GPL of the Mabc-S strain [18]. Excessive production of mitochondrial ROS, combined with hyperactivation of TNF, are presumably related to the pathogenesis of Mabc-R infection through upregulation of mixed types of cell death, including necrosis and apoptosis.

Several reagents have been reported in the modulation of SIRT3 activities in various experimental models [12]. RSV is a widely used activator for SIRT3 as well as SIRT1 and shows beneficial effects on metabolic disturbance and cardiac pathologies [34–36]. The function of RSV is, at least partly, mediated through preservation of mitochondrial functions and detoxification through deacetylation of essential mitochondrial proteins [49]. There have been numerous studies of host-directed therapeutics against human tuberculosis to accomplish better clinical outcomes through modulation of antimicrobial host defensive mechanisms as well as controlling deleterious inflammation [50–52]. SIRT is a potential host target in core regulatory pathways for targeted therapy against Mtb [51]. Several previous studies reported an inhibitory effect of RSV or RSV derivatives on Mtb growth [53–55]. Mechanistically, the protective effect of RSV is mediated through activation of autophagy and amelioration of inflammatory responses during Mtb infection [55]. Compared with tuberculosis, there are very few studies of host-directed therapeutics against NTM diseases, presumably due to a lack of host responses during NTM infections. We previously found that honokiol, a more specific agonist for SIRT3 [56], inhibited intracellular survival of Mtb and promoted mitochondrial homeostasis [16]. However, we found that honokiol led to increased cell death in macrophages during Mabc infection (data not shown). Importantly, administration of RSV was beneficial in controlling bacterial burdens in the lungs of mice and ZF infected with Mabc-R and Mabc-S. Our data show that the protective effects of RSV treatment are presumably mediated through amelioration of excessive inflammatory responses and mitochondrial ROS during Mabc infection. This is the first report to reveal the potential use of RSV for treatment of Mabc infection *in vitro* and *in vivo*. Together, these data identify a previously unappreciated role for SIRT3-mediated mitochondrial homeostasis in controlling host defenses during Mabc-R infection.

## Supplementary Material

Supplemental Material

Supplemental Material
