# Supplementary figures and images for: Sirtuin 3 is essential for host defense against Mycobacterium abscessus infection through regulation of mitochondrial homeostasis
Source: Virulence. 2020 Sep 9;11(1):1225–39. doi: 10.1080/21505594.2020.1809961 (PMC7549921; doi:10.1080/21505594.2020.1809961)

### Figure S1

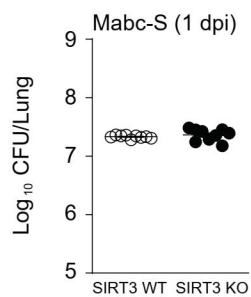

Figure S2

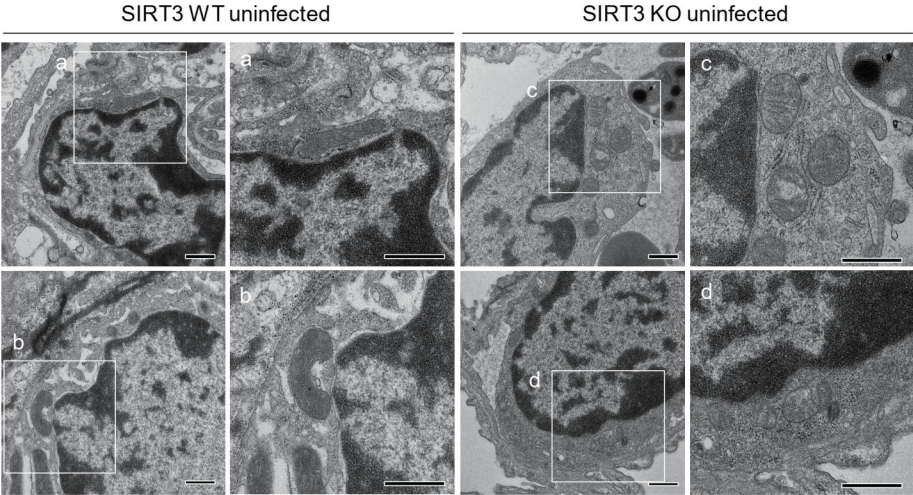

**Figure S3**

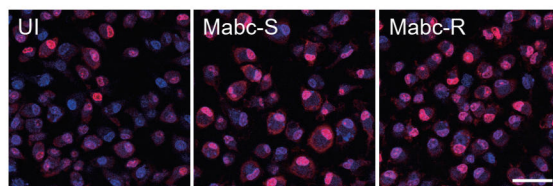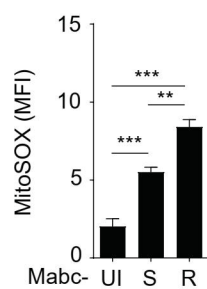

**Figure S4**

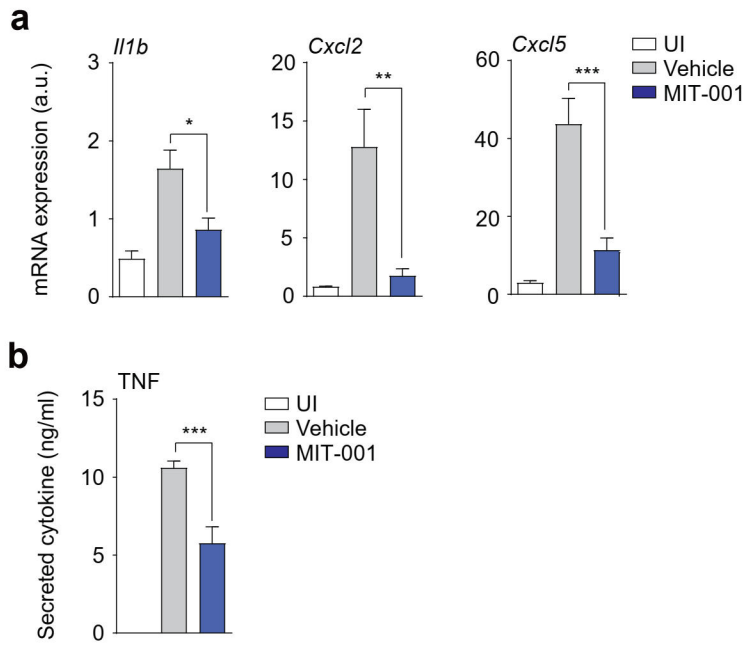

**Figure S5**

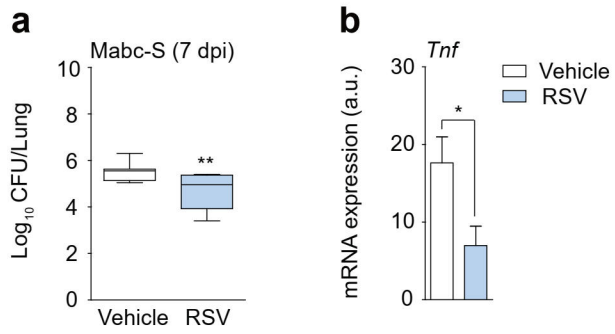

**Figure S6**

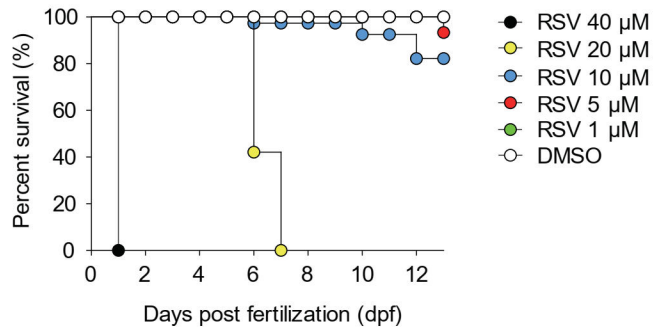

**Figure S7**

**a**

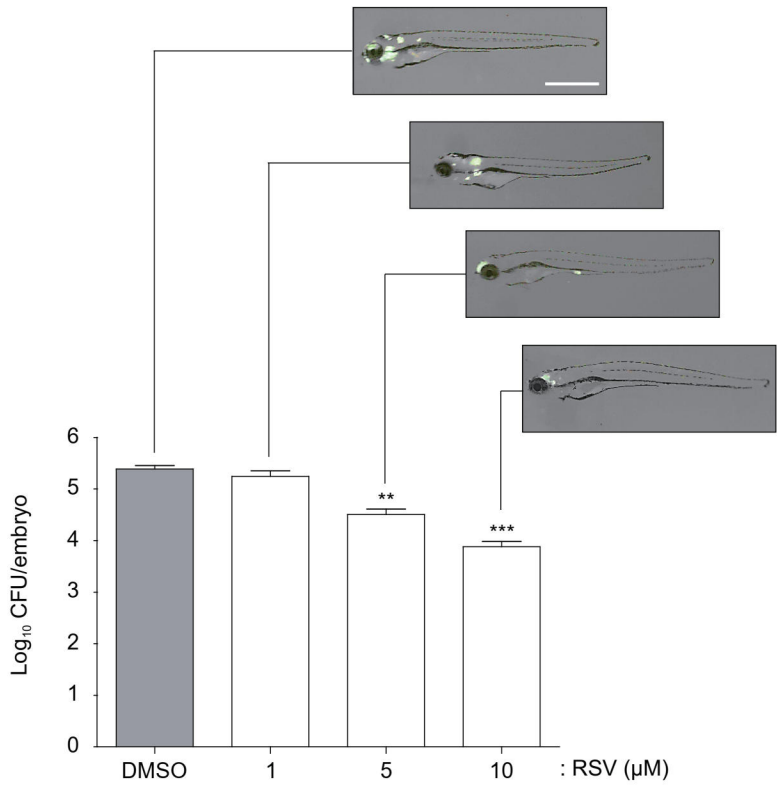

**b**

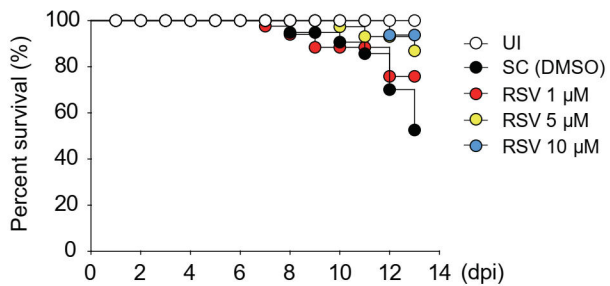

Supplement: Supplemental Material [file KVIR_A_1809961_SM1919.pdf]
