# Supplementary material for: Sirtuin 3 is essential for host defense against Mycobacterium abscessus infection through regulation of mitochondrial homeostasis
Source: Virulence. 2020 Sep 9;11(1):1225–39. doi: 10.1080/21505594.2020.1809961 (PMC7549921; doi:10.1080/21505594.2020.1809961)
Supplement: Supplemental Material [file KVIR_A_1809961_SM1071.doc]

**Supplementary Table 1.** Primers used in this study.

| Genes | Primer | Sequences |
| --- | --- | --- |
| *Tnf* | Forward  Reverse | 5′-ACGGCATGGATCTCAAAGAC-3′  5′-AGATAGCAAATCGGCTGACG-3′ |
| *Il6* | Forward  Reverse | 5′-TACCACTTCACAAGTCGGAGGC-3′  5′-CTGCAAGTGCATCATCGTTGTTC-3′ |
| *Il12p40* | Forward  Reverse | 5′-TTGAACTGGCGTTGGAAGCACG-3′  5′-CCACCTGTGAGTTCTTCAAAGGC-3′ |
| *Il1b* | Forward  Reverse | 5′-TACGGACCCCAAAAGATGA-3′  5′-TGCTGCTGCGAGATTTGAAG-3′ |
| *Ifng* | Forward  Reverse | 5′-CGGCACAGTCATTGAAAGCC-3′  5′-TGCATCCTTTTTCGCCTTGC-3′ |
| *Ccl2* | Forward  Reverse | 5′-TGACCCCAAGAAGGAATGGG-3′  5′-ACCTTAGGGCAGATGCAGTT-3′ |
| *Cxcl2* | Forward  Reverse | 5′-CCCTGCCAAGGGTTGACTTC-3′  5′-GCAAACTTTTTGACCGCCCT-3′ |
| *Cxcl5* | Forward  Reverse | 5′-CCGCTGGCATTTCTGTTGCTGT-3′  5′-CAGGGATCACCTCCAAATTAGCG-3′ |
| *Sirt3* | Forward  Reverse | 5′-GCTACATGCACGGTCTGTCGAA-3′  5′-CAATGTCGGGTTTCACAACGCC-3′ |
| *Ndufab1* | Forward  Reverse | 5′-GGACCGAGTTCTGTATGTCTTG-3′  5′-AAACCCAAATTCGTCTTCCATG-3′ |
| *Sdhb* | Forward  Reverse | 5′-ACCCCTTCTCTGTCTACCG-3′  5′-AATGCTCGCTTCTCCTTGTAG-3′ |
| *Uqcrc1* | Forward  Reverse | 5′-ATCAAGGCACTGTCCAAGG-3′  5′-TCATTTTCCTGCATCTCCCG-3′ |
| *Cox5b* | Forward  Reverse | 5′-ACCCTAATCTAGTCCCGTCC-3′  5′-CAGCCAAAACCAGATGACAG-3′ |
| *Atp5a1* | Forward  Reverse | 5′-CATTGGTGATGGTATTGCGC-3′  5′-TCCCAAACACGACAACTCC-3′ |
| *Gapdh* | Forward  Reverse | 5′-AAGATGGTGATGGGCTTCCCG-3′  5′-TGGCAAAGTGGAGATTGTTGCC-3′ |
